# Useful predictors of progression‐free survival for Japanese patients with LATITUDE‐high‐risk metastatic castration‐sensitive prostate cancer who received upfront abiraterone acetate

**DOI:** 10.1111/iju.14754

**Published:** 2021-12-04

**Authors:** Kiyoshi Takahara, Taku Naiki, Toshiki Ito, Keita Nakane, Takuya Koie, Takahiro Yasui, Hideaki Miyake, Ryoichi Shiroki

**Affiliations:** ^1^ Department of Urology School of Medicine Fujita Health University Toyoake Japan; ^2^ Department of Nephrology Nagoya City University Graduate School of Medical Sciences Nagoya Japan; ^3^ Department of Urology Hamamatsu University School of Medicine Hamamatsu Japan; ^4^ Department of Urology Gifu University Gifu Japan

**Keywords:** abiraterone acetate, LATITUDE‐high‐risk, metastatic castration‐resistant prostate cancer

## Abstract

**Objective:**

Recently, hormonal therapy using abiraterone acetate, a second‐generation androgen receptor axis‐targeted agent, was reported to improve overall survival and progression‐free survival in men with LATITUDE‐high‐risk metastatic castration‐sensitive prostate cancer. This observational multicenter study aimed to assess the efficacy of upfront abiraterone acetate in Japanese patients with LATITUDE‐high‐risk metastatic castration‐sensitive prostate cancer.

**Methods:**

The present study included 112 Japanese patients with LATITUDE‐high‐risk metastatic castration‐sensitive prostate cancer who received upfront abiraterone acetate at four institutions belonging to the Tokai Urologic Oncology Research Seminar group, between January 2018 and September 2020. Progression‐free survival and overall survival were assessed, and Cox regression analyses were carried out to evaluate the prognostic significance of upfront abiraterone acetate for progression‐free survival.

**Results:**

Within a median follow‐up period of 13 months, the progression‐free survival and overall survival rates were 76.8% and 89.3%, respectively. Both univariate and multivariable Cox regression analyses showed that the presence of Gleason pattern 5, performance status and hemoglobin were independent predictors of progression‐free survival. The patients were subsequently divided into three groups as follows: group 1, 17 patients negative for these three independent progression‐free survival predictors; group 2, 49 patients with one positive independent progression‐free survival predictor; and group 3, 45 patients with two or three independent progression‐free survival predictors. Progression‐free survival was significantly different among these three groups (*P* < 0.001).

**Conclusion:**

Upfront abiraterone acetate might provide satisfactory outcomes for Japanese patients with LATITUDE‐high‐risk metastatic castration‐sensitive prostate cancer. Gleason pattern 5, performance status and hemoglobin are potential predictors of progression‐free survival in Japanese patients with LATITUDE‐high‐risk metastatic castration‐sensitive prostate cancer who received upfront abiraterone acetate.

**Abbreviations & Acronyms**
Abi: abiraterone acetate
ADT: androgen deprivation therapy
AE: adverse event
ALP: alkaline phosphatase
Apa: apalutamide
ARAT: androgen receptor axis‐targeted
CRPC: castration‐resistant prostate cancer
ECOG: Eastern Cooperative Oncology Group
Enz: enzalutamide
Hb: hemoglobin
mCRPC: metastatic castration‐resistant prostate cancer
mCSPC: metastatic castration‐sensitive prostate cancer
OS: overall survival
PFS: progression‐free survival
PS: performance status
PSA: prostate‐specific antigen

## Background

Most mCSPC patients have an initial response to ADT. However, the majority of these patients progress to CRPC within a median of approximately 1 year.^1^, ^2^, ^3^ This progression to CRPC is most commonly driven by reactivation of androgen receptor signaling.^4^

ADT plus docetaxel has been widely accepted as one of the standard treatments for patients with mCSPC who are eligible for chemotherapy, especially those with a high metastatic burden.^5^, ^6^, ^7^ Recently, second‐generation ARAT agents were added to ADT, including Abi, Enz or Apa. These have been shown to significantly benefit patients with mCSPC compared with ADT monotherapy.^8^, ^9^, ^10^, ^11^ Among them, hormonal therapy using Abi, which is the prodrug of abiraterone and inhibits cytochrome P‐450c17, a critical enzyme in androgen biosynthesis,^12^, ^13^ was reported to improve OS and radiographic PFS in men with high‐risk mCSPC who show at least two of the following factors: (i) Gleason score ≥8; (ii) at least three bone lesions; and (iii) the presence of visceral metastasis (LATITUDE criteria).^11^ Enz with ADT significantly reduced the risk of metastatic progression or death overtime versus placebo plus ADT in men with mCSPC, including those with low‐volume disease and/or prior docetaxel.^8^ In the TITAN clinical trials of Apa, OS and radiographic PFS were significantly longer when Apa was added to ADT than that with placebo plus ADT. Furthermore, the side‐effect profile did not differ substantially between the two groups.^9^ However, as these recent introductions of upfront ARAT agents could produce certain benefits for mCSPC patients, current therapeutic strategies for them have become markedly complex, occasionally resulting in difficulties in decision‐making in real‐world clinical practice.

Considering these findings, it is necessary to understand the prognostic factors of upfront ARAT agents for patients with mCSPC. Therefore, in the current study, we focused on upfront Abi use in LATITUDE‐high‐risk Japanese mCSPC patients. We retrospectively analyzed the prognostic outcomes of Japanese patients with LATITUDE‐high‐risk mCSPC who received upfront Abi and developed a novel system to stratify the prognosis of these patients.

## Methods

In the current study, we retrospectively analyzed the clinical data of 112 Japanese patients with LATITUDE‐high‐risk mCSPC who received upfront Abi between January 2018 and September 2020 at four institutions belonging to the Tokai Urologic Oncology Research Seminar group, including the Fujita Health University School of Medicine, Nagoya City University Graduate School of Medical Sciences, Hamamatsu University School of Medicine and Gifu University. The design of this study was approved by the ethics committee of these four institutions (approval no: HM20‐465, 60‐21‐0018, 2021‐042, 21‐051). The requirement for informed consent from all patients included in this study was waived because of the retrospective design.

In the present study, all the patients fulfilled the LATITUDE criteria, which means patients had at least two of the following factors: (i) Gleason score ≥8; (ii) at least three bone lesions; (iii) and the presence of visceral metastasis. The patients received ADT combined with oral Abi (1000 mg once daily) + prednisolone (5 mg or 10 mg once daily). Baseline assessments were carried out before the introduction of Abi. The Gleason score was obtained from the prostate biopsy. PS was assessed using the ECOG–PS, and laboratory data including initial PSA, Hb and ALP were measured using standard methods. Patients also underwent radiological examinations, including pelvic magnetic resonance imaging, computed tomography and radionuclide bone scanning, to determine the cTNM stage. Clinical, biochemical or radiographic progressive disease was defined according to the criteria of the Prostate Cancer Clinical Trials Working Group 3.

All data were analyzed using IBM spss Statistics version 23 (SPSS Japan, Tokyo, Japan). A *P*‐value of <0.05 was considered significant. OS and PFS were estimated using the Kaplan–Meier method, and differences were determined using the log‐rank test. Univariate and multivariable analyses were carried out using Cox proportional hazards regression.

## Results

The clinical characteristics of the 112 Japanese patients with LATITUDE‐high‐risk mCSPC who received upfront Abi included in the present study are summarized in Table 1.

**Table 1:** Baseline patient characteristics

Variables	*n* = 112
Median age, years (IQR)	74 (68–79)
Median initial PSA, ng/mL (IQR)	291.9 (77.9–1265.0)
Gleason score (%)	
4 + 3	1 (0.9)
4 + 4	38 (33.9)
5 + 3	1 (0.9)
4 + 5	37 (33)
5 + 4	21 (18.8)
5 + 5	13 (11.6)
Unknown	1 (0.9)
cT (%)	
2a	4 (3.6)
2b	3 (2.7)
2c	13 (11.6)
3a	26 (23.2)
3b	31 (27.7)
4	35 (31.3)
cN (%)	
0	50 (44.6)
1	62 (55.4)
No. bone metastasis, *n* (%)	
0	7 (6.3)
1 or 2	5 (4.5)
≥3	100 (89.3)
Use of bone‐modifying agents, *n* (%)	70 (62.5)
Visceral metastasis, *n* (%)	
None	71 (63.4)
Lung	36 (32.1)
Liver	2 (1.8)
Others	3 (2.7)
ECOG PS, *n* (%)	
0	86 (76.8)
1	12 (10.7)
2	12 (10.7)
3	2 (1.8)
Hb, g/dL, median (IQR)	13.4 (11.7–14.5)
ALP, IU/mL, median (IQR)	450.5 (262–1148)
Local radiation therapy, *n* (%)	2 (1.8)
Subsequent therapy, *n* (%)	
None	82 (73.2)
Enzalutamide	12 (10.7)
Bicalutamide	3 (2.7)
Flutamide	1 (0.9)
Docetaxel	8 (7.1)
Others	6 (5.4)

**Fig. 1:**
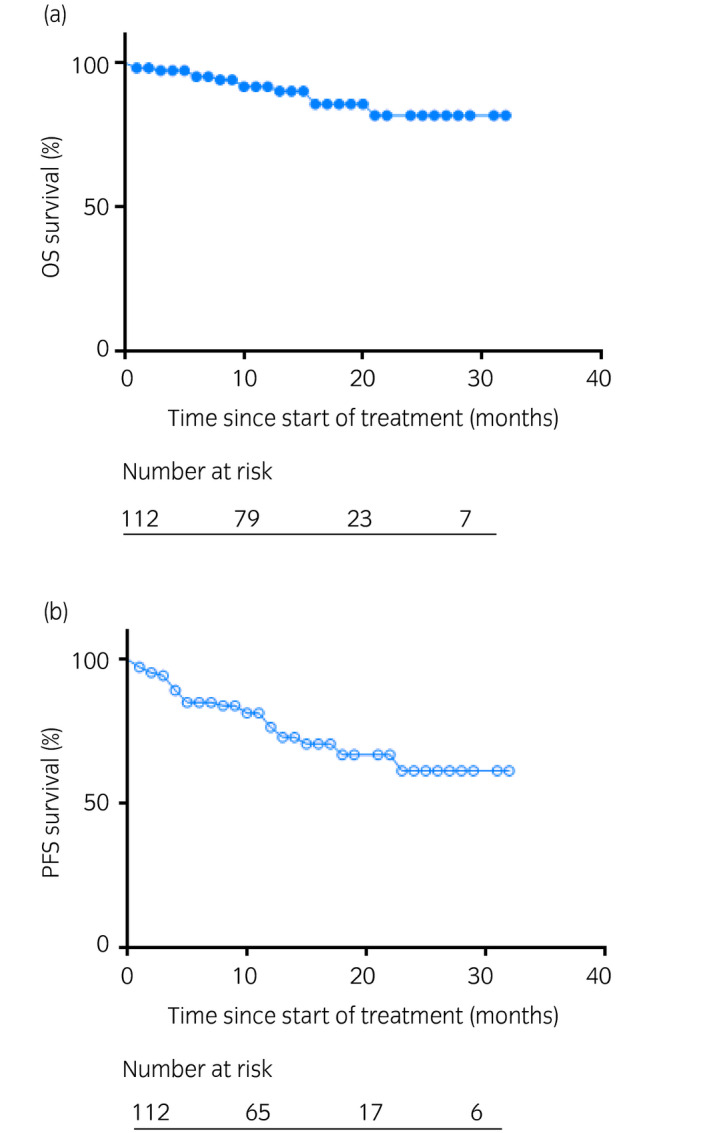
Kaplan–Meier curves of (a) OS and (b) PFS.

During the observation period with a median follow‐up period of 13 months, the OS and PFS rates were 89.3% and 76.8%, respectively (Fig. 1).

All AEs appeared in 32 patients (28.6%). Detailed information about the AEs is shown in Table S1.

To investigate the important factors affecting PFS in Japanese patients with LATITUDE‐high‐risk mCSPC who received upfront Abi, Cox regression analysis was carried out. In the univariate analysis, Gleason pattern 5, PS and Hb were identified as important predictors of PFS (*P* = 0.033, 0.004 and 0.006, respectively). Multivariable analysis using these three important predictors showed that all of these factors were independently associated with PFS (Gleason pattern 5: HR 0.235, 95% CI 0.077–0.714, *P* = 0.011; PS: HR 0.223, 95% CI 0.098–0.506, *P* = 0.000; Hb: HR 0.269, 95% CI 0.113–0.644, *P* = 0.003, respectively; Table 2).

**Table 2:** Univariate and multivariable analyses of the clinical parameters of PFS

	Univariate analysis		Multivariable analysis	
	HR (95% CI)	*P*‐value	HR (95% CI)	*P*‐value
Age (≥74 *vs* <74 years)	0.525 (0.233–1.183)	0.120		
Initial PSA (≥291.9 *vs* <291.9 ng/mL)	1.708 (0.772–3.778)	0.186		
Existence of Gleason pattern 5 (yes *vs* no)	0.312 (0.107–0.909)	0.033	0.235 (0.077–0.714)	0.011
Clinical stage cT2		0.936		
cT3	1.214 (0.396–3.714)	0.735		
cT4	1.022 (0.423–2.467)	0.962		
cN (+ *vs* −)	0.663 (0.295–1.489)	0.319		
No. bone metastasis				
0		0.286		
1 or 2	2.365 (0.814–6.869)	0.114		
≥3	–	0.976		
Visceral metastasis none		0.921		
Lung	0.695 (0.092–5.261)	0.724		
Liver	0.566 (0.070–4.556)	0.592		
Others	0.891 (0.055–14.453)	0.935		
ECOG PS (≥1 *vs* 0)	0.313 (0.143–0.683)	0.004	0.223 (0.098–0.506)	0.000
Hb (<13.1 [*n* = 50] *vs* ≥13.1 [*n* = 62] g/dL)	0.293 (0.123–0.698)	0.006	0.269 (0.113–0.644)	0.003
ALP (≥350 [*n* = 66] *vs* <350 [*n* = 46] IU/mL)	0.491 (0.206–1.170)	0.108		
Local radiation therapy (yes *vs* no)	20.752 (0.000–5639103)	0.635		

**Fig. 2:**
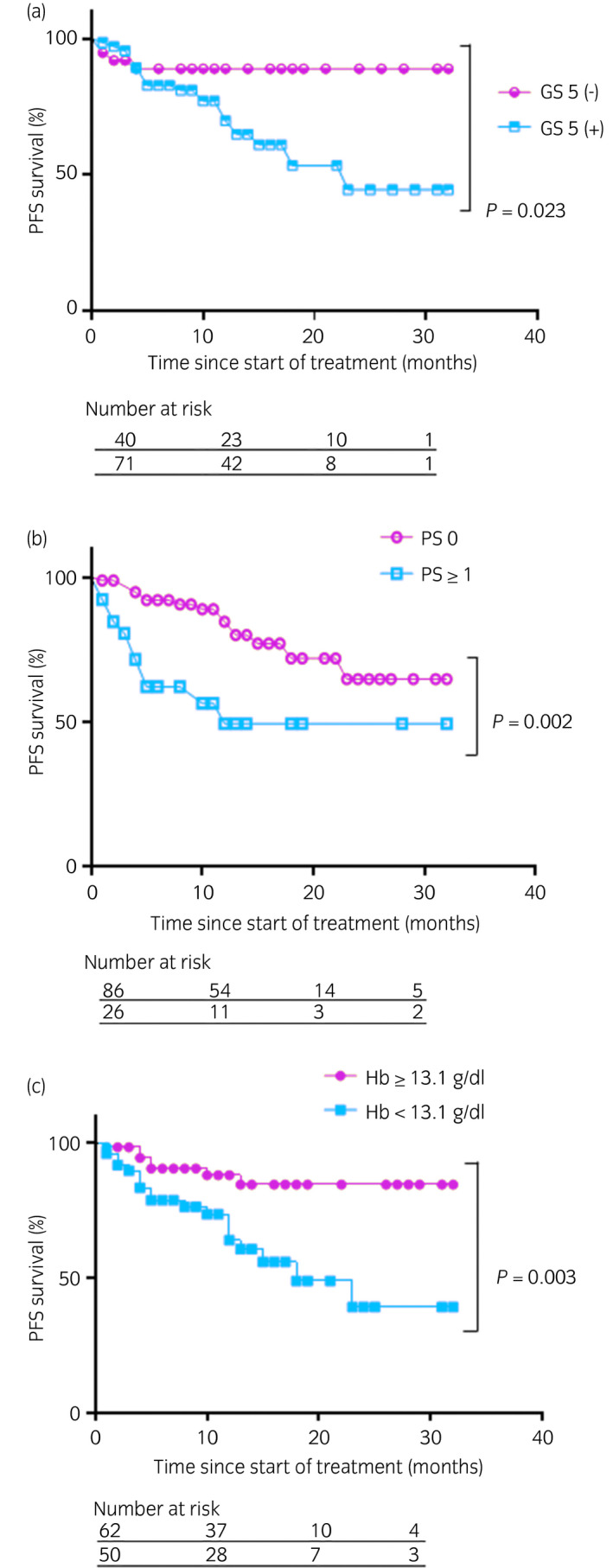
Kaplan–Meier curves for PFS and (a) Gleason score 5, (b) PS and (c) Hb.

Regarding the Gleason pattern 5, PS and Hb, Kaplan–Meier curves of PFS are shown in Fig. 2. In each category, a significant difference in PFS was observed (Gleason pattern 5, PS and Hb; *P* = 0.023, 0.002 and 0.003, respectively).

**Fig. 3:**
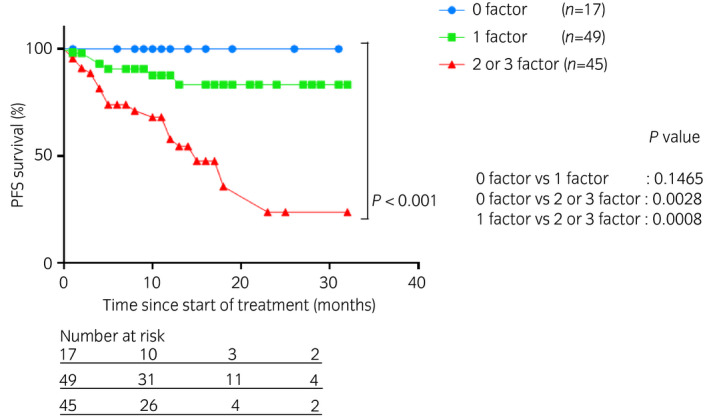
Kaplan–Meier curves for PFS according to the number of independent PFS predictors.

Considering the three independent predictors of PFS, we stratified the 111 patients into three groups as follows: group 1, 17 patients negative for these three independent PFS predictors; group 2, 49 patients with one positive independent PFS predictor; and group 3, 45 patients with two or three independent PFS predictors. The PFS was significantly different among the three groups (*P* < 0.001; 0 factor *vs* 1 factor: *P* = 0.1465; 0 factor *vs* 2 or 3 factors: *P* = 0.0028; 1 factor *vs* 2 or 3 factors: *P* = 0.0008, respectively; Fig. 3).

## Discussion

According to the extent of metastatic disease and presence of symptoms, the estimated mean survival of mCRPC patients was 9–36 months.^14^ Despite recent introductions, such as ARAT, docetaxel or cabazitaxel, the prognosis of these patients after the mCRPC stage has not dramatically improved. Accordingly, it is necessary to provide optimal treatment for the mCSPC stage and delay the mCRPC stage for as long as possible. Several recent investigators have advocated the time from diagnosis to CRPC as a significant prognosticator of OS.^15^, ^16^, ^17^ Regarding the treatment of mCSPC patients, according to the network meta‐analysis, ADT plus Abi or Apa might provide the largest OS benefits, with relatively low serious AE risks. Enz might improve radiographic PFS to the greatest extent, but a longer follow up is required to examine the OS benefits associated with Enz.^18^ Recently, Harada *et al*. showed a possible treatment strategy for patients with mCSPC as per cancer and patient characteristics, as well as patient preference.^19^ However, to date, the prognostic factors of upfront ARAT agents for Japanese patients with mCSPC have not been widely introduced into real‐world clinical practice. Collectively, further prognostication should be carried out to provide more precise information regarding the treatment of Japanese patients with mCSPC. For the mCSPC stage, current decision‐making in real‐world clinical practice should focus on Abi, which is an ARAT agent for upfront use in LATITUDE‐high‐risk Japanese mCSPC patients.

In the current study, we retrospectively analyzed the data of 112 Japanese patients with LATITUDE‐high‐risk mCSPC who received upfront Abi. Both univariate and multivariable Cox regression analyses showed that the presence of the Gleason pattern 5, PS and Hb were independent predictors of PFS. Regarding the Gleason pattern 5, prostate cancer with a Gleason score 9–10 is indicative of particularly aggressive disease.^20^, ^21^, ^22^ PS as a prognostic factor for OS has been evaluated in men with CRPC.^23^ Furthermore, several recent investigators have advocated that anemia is a powerful prognostic factor in PC.^24^, ^25^ In particular, Okamoto *et al*. reported that pretreatment anemia was an independent prognostic factor that predicted oncological outcomes among mCSPC patients treated with ADT monotherapy or complete androgen blockade.^25^ Regarding the prognostic factor of upfront Abi used for LATITUDE‐high‐risk mCSPC patients, a recent study reported pretreatment anemia was a prognostic factor among mCSPC patients who received upfront Abi.^26^

To properly predict the clinical outcomes of Japanese mCSPC patients who receive upfront Abi, we attempted to develop a novel system for the prognostic stratification of these patients by using three independent PFS predictors, the Gleason pattern 5, PS and Hb. We divided the patients into three groups based on the presence of none, one and two or three independent PFS predictors. We then compared PFS among the three groups. The results of the present study supported our novel stratification system, suggesting that the positive numbers of independent PFS predictors could be a useful tool for the treatment of Japanese patients with LATITUDE‐high‐risk mCSPC who receive upfront Abi.

The present study had several limitations. First, this was a retrospective study with a small sample size and a short‐term follow‐up period. In particular, because of the short observation period with a median follow‐up period of 13 months, proper analyses assessing prognostic factors for OS could not be carried out considering the high OS rates. Second, the cut‐off points used in the current analyses should be assessed in a large‐scale study. Third, we could not obtain sufficient patient information, including their comorbidities, past history and the extent of disease of bone metastasis. Future prospective studies with much larger sample sizes and longer follow‐up periods are required to confirm the findings of the current study.

In conclusion, we identified that upfront Abi might provide satisfactory clinical outcomes for Japanese patients with LATITUDE‐high‐risk mCSPC. The Gleason pattern 5, PS and Hb levels might be considered as useful predictors of PFS in these patients. Furthermore, our novel stratification system based on the positive numbers of these three independent PFS predictors could help guide decision‐making for the treatment of Japanese patients with LATITUDE‐high‐risk mCSPC who receive upfront Abi.

## Conflict of interest

None declared.

## Approval of the research protocol by an Institutional Reviewer Board

The design of this study was approved by the ethics committee of the four institutions (approval no: HM20‐465 [Fujita Health University School of Medicine], 60‐21‐0018 [Nagoya City University Graduate School of Medical Sciences], 2021‐042 [Hamamatsu University School of Medicine], 21‐051 [Gifu University]).

## Informed consent

Not applicable.

## Registry and the Registration No. of the study/trial

Not applicable.

## Animal studies

Not applicable.

## Supporting information


**Table S1**. Adverse events.

## References

[1] Gravis G , Fizazi K , Joly F *et al*. Androgen‐deprivation therapy alone or with docetaxel in non‐castrate metastatic prostate cancer (GETUG‐AFU 15): a randomised, open‐label, phase 3 trial. Lancet Oncol. 2013; 14: 149–58. 23306100 10.1016/S1470-2045(12)70560-0

[2] James ND , Spears MR , Clarke NW *et al*. Survival with newly diagnosed metastatic prostate cancer in the “Docetaxel Era”: data from 917 patients in the control arm of the STAMPEDE trial (MRC PR08, CRUK/06/019). Eur. Urol. 2015; 67: 1028–38. 25301760 10.1016/j.eururo.2014.09.032

[3] Sweeney CJ , Chen Y‐H , Carducci M *et al*. Chemohormonal therapy in metastatic hormone‐sensitive prostate cancer. N. Engl. J. Med. 2015; 373: 737–46. 26244877 10.1056/NEJMoa1503747 PMC4562797

[4] Watson PA , Arora VK , Sawyers CL . Emerging mechanisms of resistance to androgen receptor inhibitors in prostate cancer. Nat. Rev. Cancer 2015; 15: 701–11. 26563462 10.1038/nrc4016 PMC4771416

[5] Cornford P , Bellmunt J , Bolla M *et al*. EAU‐ESTRO‐SIOG guidelines on prostate cancer. Part II: treatment of relapsing, metastatic, and castration‐resistant prostate cancer. Eur. Urol. 2017; 71: 630–42. 27591931 10.1016/j.eururo.2016.08.002

[6] Gillessen S , Omlin A , Attard G *et al*. Management of patients with advanced prostate cancer: recommendations of the St Gallen Advanced Prostate Cancer Consensus Conference (APCCC) 2015. Ann. Oncol. 2015; 26: 1589–604. 26041764 10.1093/annonc/mdv257 PMC4511225

[7] Parker C , Gillessen S , Heidenreich A , Horwich A , Committee EG . Cancer of the prostate: ESMO Clinical Practice Guidelines for diagnosis, treatment and follow‐up. Ann. Oncol. 2015; 26(Suppl 5): v69–77. 26205393 10.1093/annonc/mdv222

[8] Armstrong AJ , Szmulewitz RZ , Petrylak DP *et al*. ARCHES: a randomized, Phase III study of androgen deprivation therapy with enzalutamide or placebo in men with metastatic hormone‐sensitive prostate cancer. J. Clin. Oncol. 2019; 37: 2974–86. 31329516 10.1200/JCO.19.00799 PMC6839905

[9] Chi KN , Agarwal N , Bjartell A *et al*. Apalutamide for metastatic, castration‐sensitive prostate cancer. N. Engl. J. Med. 2019; 381: 13–24. 31150574 10.1056/NEJMoa1903307

[10] Chi KN , Protheroe A , Rodríguez‐Antolín A *et al*. Patient‐reported outcomes following abiraterone acetate plus prednisone added to androgen deprivation therapy in patients with newly diagnosed metastatic castration‐naive prostate cancer (LATITUDE): an international, randomised phase 3 trial. Lancet Oncol. 2018; 19: 194–206. 29326030 10.1016/S1470-2045(17)30911-7

[11] Fizazi K , Tran N , Fein L *et al*. Abiraterone plus Prednisone in metastatic, castration‐sensitive prostate cancer. N. Engl. J. Med. 2017; 377: 352–60. 28578607 10.1056/NEJMoa1704174

[12] Barrie SE , Potter GA , Goddard PM , Haynes BP , Dowsett M , Jarman M . Pharmacology of novel steroidal inhibitors of cytochrome P450(17) alpha (17 alpha‐hydroxylase/C17‐20 lyase). J. Steroid Biochem. Mol. Biol. 1994; 50: 267–73. 7918112 10.1016/0960-0760(94)90131-7

[13] O'Donnell A , Judson I , Dowsett M *et al*. Hormonal impact of the 17alpha‐hydroxylase/C(17,20)‐lyase inhibitor abiraterone acetate (CB7630) in patients with prostate cancer. Br. J. Cancer 2004; 90: 2317–25. 15150570 10.1038/sj.bjc.6601879 PMC2409523

[14] Heidenreich A , Bastian PJ , Bellmunt J *et al*. EAU guidelines on prostate cancer. Part II: treatment of advanced, relapsing, and castration‐resistant prostate cancer. Eur. Urol. 2014; 65: 467–79. 24321502 10.1016/j.eururo.2013.11.002

[15] Frees S , Akamatsu S , Bidnur S *et al*. The impact of time to metastasis on overall survival in patients with prostate cancer. World J. Urol. 2018; 36: 1039–46. 29488095 10.1007/s00345-018-2236-4

[16] Miyake H , Matsushita Y , Watanabe H *et al*. Prognostic significance of time to castration resistance in patients with metastatic castration‐sensitive prostate cancer. Anticancer Res. 2019; 39: 1391–6. 30842173 10.21873/anticanres.13253

[17] Wenzel M , Preisser F , Hoeh B *et al*. Impact of time to castration resistance on survival in metastatic hormone sensitive prostate cancer patients in the Era of combination therapies. Front. Oncol. 2021; 11: 659135. 33968764 10.3389/fonc.2021.659135 PMC8103198

[18] Wang L , Paller CJ , Hong H , De Felice A , Alexander GC , Brawley O . Comparison of systemic treatments for metastatic castration‐sensitive prostate cancer: a systematic review and network meta‐analysis. JAMA Oncol. 2021; 7: 412–20. 33443584 10.1001/jamaoncol.2020.6973 PMC7809610

[19] Harada K , Shiota M , Minato A *et al*. Treatment strategies for metastatic castration‐sensitive prostate cancer: from “All‐Comers” to “Personalized” approach. Onco. Targets Ther. 2021; 14: 2967–74. 33981146 10.2147/OTT.S306345 PMC8107048

[20] Epstein JI , Egevad L , Amin MB *et al*. The 2014 International Society of Urological Pathology (ISUP) Consensus Conference on Gleason grading of prostatic carcinoma: definition of grading patterns and proposal for a new grading system. Am. J. Surg. Pathol. 2016; 40: 244–52. 26492179 10.1097/PAS.0000000000000530

[21] Epstein JI , Zelefsky MJ , Sjoberg DD *et al*. A contemporary prostate cancer grading system: a validated alternative to the Gleason Score. Eur. Urol. 2016; 69: 428–35. 26166626 10.1016/j.eururo.2015.06.046 PMC5002992

[22] Ham WS , Chalfin HJ , Feng Z *et al*. New prostate cancer grading system predicts long‐term survival following surgery for Gleason Score 8–10 prostate cancer. Eur. Urol. 2017; 71: 907–12. 27876305 10.1016/j.eururo.2016.11.006

[23] Chen WJ , Kong DM , Li L . Prognostic value of ECOG performance status and Gleason score in the survival of castration‐resistant prostate cancer: a systematic review. Asian J. Androl. 2021; 23: 163–9. 33159024 10.4103/aja.aja_53_20 PMC7991808

[24] Mori K , Janisch F , Mostafaei H *et al*. Prognostic value of hemoglobin in metastatic hormone‐sensitive prostate cancer: a systematic review and meta‐analysis. Clin. Genitourin. Cancer 2020; 18: e402–9. 32007439 10.1016/j.clgc.2019.12.002

[25] Okamoto T , Hatakeyama S , Narita S *et al*. Impact of nutritional status on the prognosis of patients with metastatic hormone‐naive prostate cancer: a multicenter retrospective cohort study in Japan. World J. Urol. 2019; 37: 1827–35. 30511214 10.1007/s00345-018-2590-2

[26] Okamoto T , Noro D , Hatakeyama S *et al*. Impact of pretreatment anemia on upfront abiraterone acetate therapy for metastatic hormone‐sensitive prostate cancer: a multicenter retrospective study. BMC Cancer 2021; 21: 605. 34034691 10.1186/s12885-021-08206-8 PMC8152305

