# Supplementary material for: Useful predictors of progression‐free survival for Japanese patients with LATITUDE‐high‐risk metastatic castration‐sensitive prostate cancer who received upfront abiraterone acetate
Source: Int J Urol. 2021 Dec 4;29(3):229–34. doi: 10.1111/iju.14754 (PMC9299872; doi:10.1111/iju.14754)
Supplement: Supplementary file 1 — Table S1. Adverse events. [file IJU-29-229-s001.docx]

**Supplemental Table 1**

| **Incidence of AEs, n (%)** | **32 (28.6)** | | |  |
| --- | --- | --- | --- | --- |
|  | **Grade 1** | **Grade 2** | **Grade 3** | **Grade 4** |
| **Type of AEs, (%)** |  |  |  |  |
| **Hot flush** | **3 (2.7)** | **0 (0)** | **0 (0)** | **0 (0)** |
| **AST/ ALT elevation** | **9 (8.0)** | **2 (1.8)** | **8 (7.1)** | **1 (0.9)** |
| **Hypokalemia** | **3 (2.7)** | **1 (0.9)** | **2 (1.8)** | **0 (0)** |
| **Fatigue** | **5 (4.5)** | **1 (0.9)** | **2 (1.8)** | **0 (0)** |
| **Others** | **2 (1.8)** | **3 (2.7)** | **1 (0.9)** | **0 (0)** |
